# Latent Reactivation of Osteoarticular Tuberculosis of the Knee

**DOI:** 10.7759/cureus.98873

**Published:** 2025-12-10

**Authors:** Siddharth Kotikalapudi, Preetam Upadhyaya, Krishna Vemulapalli

**Affiliations:** 1 ENT, Barking, Havering and Redbridge University Hospitals NHS Trust, Romford, GBR; 2 Trauma and Orthopaedics, Barking, Havering and Redbridge University Hospitals NHS Trust, Romford, GBR

**Keywords:** chronic bone infection, extra-pulmonary tb, knee trauma, orthopaedics surgery, osteo-articular tb

## Abstract

Osteoarticular tuberculosis (TB) is an uncommon manifestation of extrapulmonary TB (EPTB), accounting for approximately 10-15% of such cases. Amongst these, knee involvement is particularly rare, representing only 0.1-0.3% of all TB infections. Due to its rarity and indolent presentation, tuberculous arthritis of the knee can be misdiagnosed as pyogenic septic arthritis or degenerative joint disease. We report the case of a 43-year-old Indian woman, who has been a resident in the United Kingdom for eight years, who presented with a two-week history of progressive swelling of the left knee that spontaneously discharged thick, caseous material. She had sustained a minor fall onto the same knee five months earlier but had no systemic symptoms or prior history of TB. Imaging demonstrated chronic inflammatory changes, and intraoperative samples from sinus excision and joint washout tested positive for *Mycobacterium tuberculosis* on acid-fast bacilli (AFB) smear and polymerase chain reaction (PCR). Further systemic evaluation revealed no evidence of pulmonary or disseminated disease. She was commenced on standard anti-tuberculous therapy and made a good postoperative recovery. This case illustrates an uncommon presentation of knee TB likely resulting from trauma-induced reactivation of latent infection. Trauma may create a pro-inflammatory, hyperaemic microenvironment that disrupts local immune control, allowing dormant bacilli to proliferate. Diagnosis requires a high index of suspicion, particularly in patients from endemic regions. Imaging assists in defining disease extent, whilst confirmation rests on microbiological and molecular testing. Combined surgical and medical management remains the cornerstone of therapy. Tuberculous arthritis should be considered in the differential diagnosis of chronic monoarthritis, even in the absence of systemic features or pulmonary involvement. Early multidisciplinary assessment and prompt initiation of anti-tuberculous therapy are essential to preserve joint function and prevent disability.

## Introduction

Extrapulmonary tuberculosis (EPTB) accounts for approximately 15-20% of all TB cases in immunocompetent individuals [1], and this proportion can rise to 50% or more in immunosuppressed patients [2]. Amongst EPTB cases, osteoarticular TB is relatively uncommon, representing around 10-15% of all EPTB presentations, whilst lymph nodes and pleura remain the most frequent sites [2].

Pott’s disease (TB of the spine) is the most common manifestation of osteoarticular TB, with the hips and knees being the second most frequently affected joints [3]. Consequently, presentations of TB involving the knee are rare, at just 0.1-0.3% in all forms of TB [4]. Due to its rarity, knee TB can be misdiagnosed as pyogenic septic arthritis, osteoarthritis, or post-traumatic arthritis.

This case study discusses an unusual presentation of mono-articular TB in an Indian migrant, who presented with knee swelling and subsequent discharge. She had no preceding systemic TB symptoms or red-flag signs. However, she had sustained a fall onto her left knee five months prior to presentation, which is inferred to be the cause of latent TB reactivation (in light of no interferon-gamma release assay (IGRA)/Mantoux or prior demonstration of TB focus) [2]. This presentation highlights the importance of tailoring the differential diagnosis to patient factors such as ethnicity and exposure to atypical pathogens from endemic regions

## Case presentation

A 43-year-old Indian woman, resident in the United Kingdom for the past eight years, presented with a two-week history of progressive swelling of the left knee. The swelling spontaneously ruptured, discharging thick, cottage cheese-like material. She reported a minor fall onto the same knee approximately five months earlier but had otherwise been in good health with no significant past medical history. She denied constitutional symptoms such as fever, weight loss, or night sweats. There was no history of recent infection, joint disease, or tuberculosis exposure. She had received the Bacillus Calmette-Guérin (BCG) vaccination as a child.

On examination, there was a 0.5 × 0.5 cm sinus over the anteromedial aspect of the left knee with active purulent discharge. There was mild peri-sinus erythema but no gross deformity. Knee flexion was limited to 30 degrees actively and 40 degrees passively. Straight-leg raise on the left side was normal, and she was able to fully weight-bear. There was no palpable lymphadenopathy, and chest and cardiovascular examinations were unremarkable.

**Figure 1:**
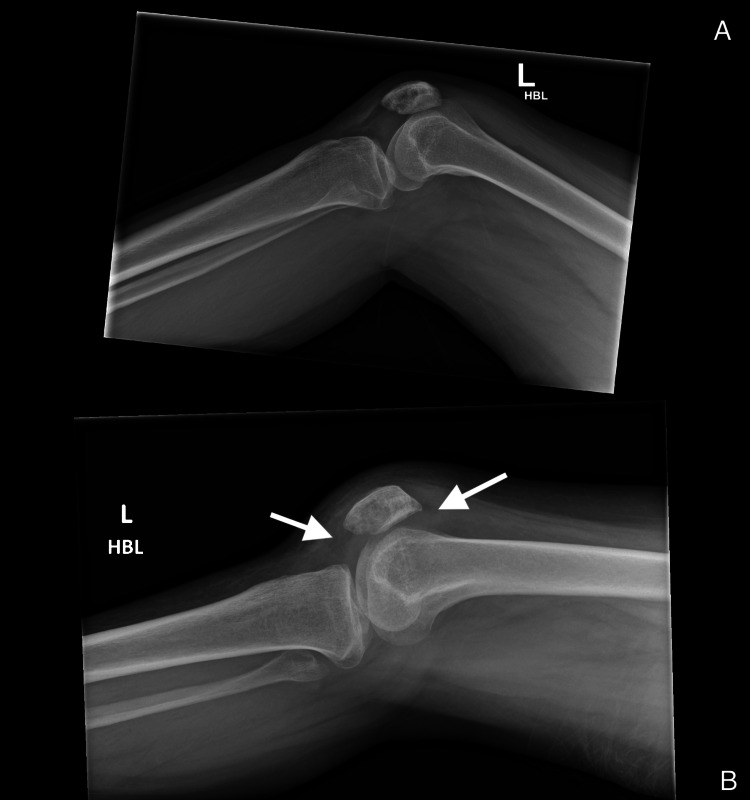
Radiographs of her left knee. (A) Five months prior to her initial presentation. (B) Five months post-fall and upon initial presentation to the orthopaedic team (A) No evidence for any cortical disruption or a joint effusion suggestive of fracture or soft-tissue injury. (B) Five months after her initial fall. The lateral knee radiograph shows a small effusion, which is highlighted by the arrows

**Figure 2:**
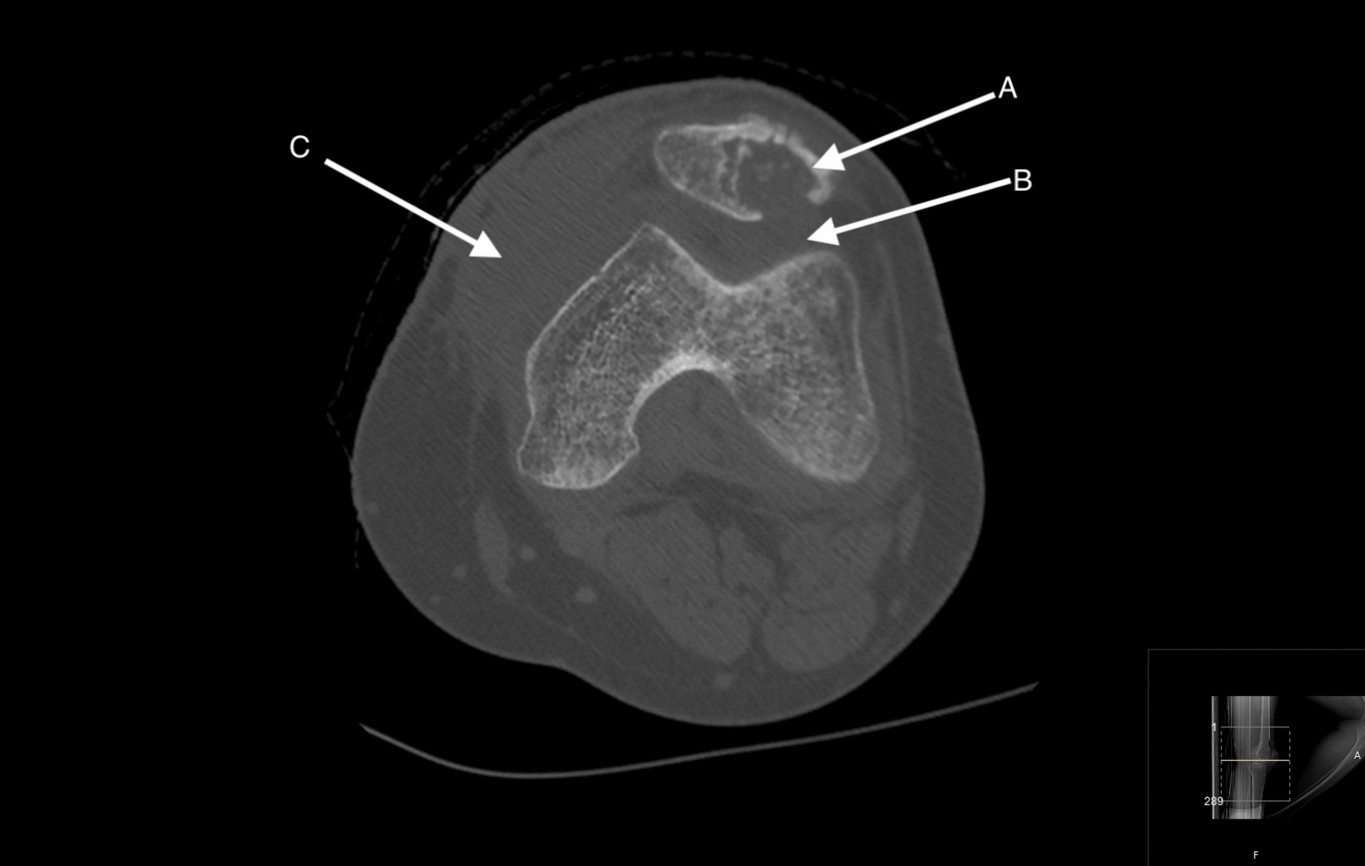
Axial CT scan of the left knee in bone window (A)  Large well-defined lytic lesion (25 x 17 mm) with cortical thinning and scalloping. (B) Extension of this lesion into the infra-patellar fat pad by the formation of a collection. (C) Extra-articular extension of the collection medially and into the subcutaneous soft tissue. The large collection measures 50 x 20 mm

**Figure 3:**
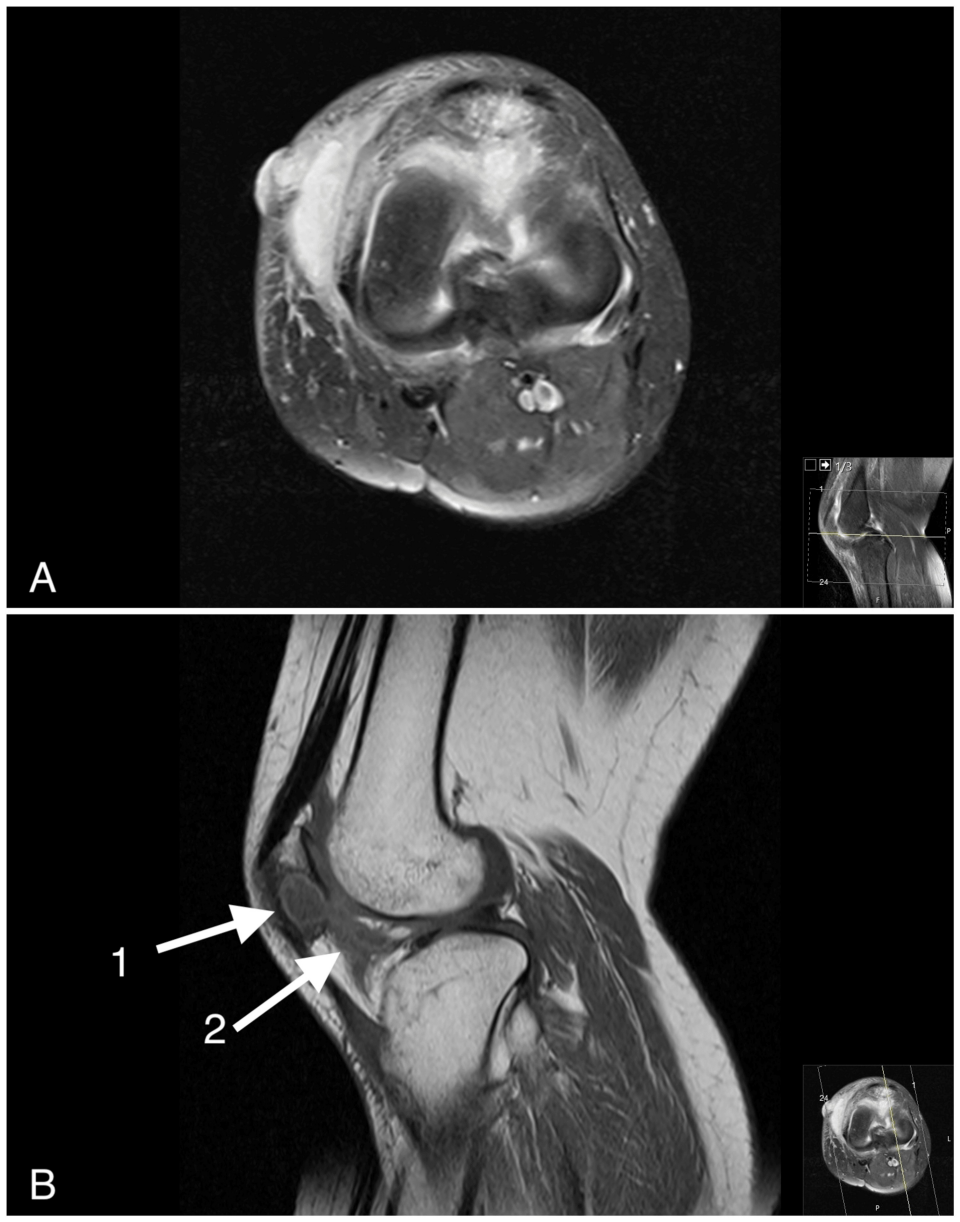
MRI of the left knee (A) Axial PD TSE view of the knee. The high signal highlights the extent of the intra-osseous collection in the patella, which is connected to the sinus tract through Hoffa's fat pad. (B) T1 sagittal view of the knee: (1) the intra-patellar lytic lesion, (2) extension into Hoffa's fat pad

Routine blood tests were largely unremarkable, with only a mildly raised C-reactive protein (CRP) (Table 1). Radiological investigations, including plain radiographs (Figure 1), CT (Figure 2) and MRI of the left knee, demonstrated features consistent with chronic inflammatory or infective change (Figure 3). The case was reviewed by a tertiary orthopaedic multidisciplinary team (MDT) to exclude malignancy before proceeding with surgery. The outcome from their MDT advised surgical intervention, as they suspected it was likely caseous necrosis secondary to TB, not malignancy.

**Table 1:** Initial results of laboratory blood tests

Lab investigation	Results	Normal reference range	Unit
White cell count	7.1	3.8-11.0	x 10^9^/L
Haemaglobin	118	115-155	g/L
Neutrophils	7.1	2-7.5	x 10^9^/L
Lymphocytes	1	1.5-4.0	x 10^9^/L
C-reactive protein	8	<5	mg/L

She underwent excision of the sinus tract and washout of the knee joint, with intraoperative samples sent for acid-fast bacilli (AFB) smear and *Mycobacterium tuberculosis* polymerase chain reaction (TB PCR). The sinus tract was excised through an elliptical incision and was confirmed intraoperatively to communicate with the knee joint. Unhealthy granulation tissue was noted throughout the sinus tract and within the joint and was completely excised. The knee joint was then thoroughly irrigated with 9 litres of normal saline and closed in layers using 1-0 Vicryl for deep tissues and 2-0 nylon for the skin. Both the AFB smear and TB PCR tests returned positive results, confirming the diagnosis of tuberculous arthritis of the knee. Subsequent discussion with infectious diseases and respiratory medicine teams led to further evaluation with CT chest, abdomen, and pelvis, which revealed no evidence of pulmonary or disseminated tuberculosis but identified an incidental left adnexal cyst, later confirmed to be an ovarian cyst on transvaginal ultrasound.

The patient was commenced on anti-tuberculous chemotherapy (rifater 6 tablets OD, ethambutol 1 gm OD, pyridoxine 20 mg OD) and discharged with outpatient follow-up. At follow-up at one month post-surgery, she was recovering well, fully weight-bearing, and mobilising independently with gradual improvement in range of motion. She will require long-term follow-up to monitor functional recovery and exclude recurrence.

## Discussion

TB remains one of the leading causes of death worldwide, with an estimated 10.8 million people developing TB in 2023, corresponding to a global incidence rate of 134 per 100,000 population. The geographic distribution of TB is uneven: the Southeast Asia region accounts for 45% of cases, Africa with 24% (with the highest burden of HIV-associated TB), the Western Pacific with 17%, the Eastern Mediterranean with 8.6%, and the Americas and Europe together with approximately 5.3%, with higher prevalence in Eastern Europe [5,6].

Within Europe, whilst the overall incidence of TB has been gradually decreasing, there has been an increased prevalence of EPTB. This could be due to effective screening for pulmonary TB and migration from endemic regions, who are more likely to have latent reactivation of TB [7,8]. 

Osteoarticular TB develops through haematogenous spread from a primary focus, often pulmonary or lymphatic, though in many cases this source is clinically silent. The infection begins in the synovium and gradually extends to the articular cartilage and subchondral bone, resulting in progressive pain, swelling, sinus formation, and joint stiffness [9].

In this patient, the absence of systemic symptoms and the presence of a small draining sinus could have led to a misdiagnosis as a simple abscess, septic arthritis, or an unusual case of post-traumatic infection. However, her history and risk factors (immigrant from an endemic region) raised suspicions for osteoarticular TB, prompting further investigation. Confirmatory diagnosis was achieved through positive AFB staining and TB PCR.

Although our patient had no evidence of active or prior pulmonary TB, her upbringing in an endemic region suggests previous latent infection. *Mycobacterium tuberculosis *bacilli can remain dormant for decades within the reticuloendothelial or skeletal system [10]. Local trauma has been reported to be a predisposing factor in reactivation of latent TB in multiple cases [4]; however, there has been scant research into how or why trauma predisposes to latent reactivation. 

Current research suggests that trauma triggers a localised and systemic inflammatory response that may disrupt the balance between host defenses and the latent bacteria. It is known that *Mycobacterium tuberculosis *readily infects monocytes, with the histological hallmark of disease being the formation of granulomas that are described as containing the disease. Studies have shown that these *Mycobacterium*-infected monocytes can freely migrate in and out of granulomas; thus, it is postulated that the release of chemoattractants at sites of local trauma recruits these infected monocytes and provides a rich nidus for the reactivation of the latent TB within these monocytes [11]. However, this is a proposed hypothesis from limited data and requires robust research to confirm this proposed mechanism. The increased vascularity due to reactive hyperaemia following trauma helps provide a favourable niche for dormant TB bacilli to reactivate [2]. Research regarding vascular supply or compromise following trauma and TB reactivation is limited and requires robust research to confirm the importance of vascular supply after trauma in latent reactivation. 

Imaging plays an important role in the assessment of osteoarticular TB. Early radiographic findings are often non-specific; MRI provides significant soft-tissue detail and can detect synovial thickening, joint effusion, and bone marrow changes suggestive of tuberculous involvement. In this case, an MRI of the knee was requested after an initial radiograph, as it can be considered to be the most sensitive imaging modality for early detection and soft tissue involvement [12]. CT scans were used for improved visualisation of cortical bone and sequestra [12]. The imaging findings supported a chronic infective process and guided surgical planning. 

Management of osteoarticular TB involves combined medical and surgical therapy. The cornerstone of treatment is standard anti-tuberculous chemotherapy (typically isoniazid, rifampicin, pyrazinamide, and ethambutol for two months, followed by continuation therapy with isoniazid and rifampicin for a further 4-10 months). Surgery is indicated for diagnostic biopsy, debridement, sinus excision, or when there is abscess formation or advanced joint destruction [2].

## Conclusions

Our patient underwent sinus excision and washout, followed by anti-tuberculous therapy (which she is still currently undergoing), resulting in encouraging recovery with preservation of function (will require long-term follow-up to confirm the extent of her recovery). Her case emphasises that TB should remain in the differential diagnosis of chronic joint infections, particularly in patients originating from endemic regions, even after years of residence in low-prevalence countries.
